# Potential Risks and Factors of Women’s Health Promotion

**DOI:** 10.3390/ijerph17249555

**Published:** 2020-12-21

**Authors:** Claudio Costantino, Alessandra Casuccio, Vincenzo Restivo

**Affiliations:** Department of Health Promotion, Maternal and Infant Care, Internal Medicine and Medical Specialties “G. D’Alessandro”, Section of Hygiene, University of Palermo, Via Del Vespro 133, 90127 Palermo, Italy; alessandra.casuccio@unipa.it (A.C.); vincenzo.restivo@unipa.it (V.R.)

In addition to diseases shared by both sexes, there are a number of illnesses and injuries that are primarily associated with women [1]. These health problems are less likely to be detected and treated because of the narrow framework used in considering healthcare for women that results from the lack of awareness of both the recipients and providers regarding the extent of women’s healthcare needs and their requirements for comprehensive care [2].

Indeed women are either not aware of such healthcare needs, or they are aware but tend to ignore these needs because of their demanding role responsibilities, workload, and other caregiving activities, or they have been prevented from seeking healthcare and from maintaining their health by limited resources and structural constraints [3].

The Special Issue “Potential Risks and Factors of Women’s Health Promotion” of *International Journal of Environmental Research and Public Health* was launched in June 2019 with the main aim of implementing international literature data on various aspects of women’s health, such as breastfeeding, HPV, and gynecological diseases [4]. Interdisciplinary works and multi-country collaborative research studies were especially welcomed. The submission of original articles, systematic reviews or meta-analyses, short communications, and other types of article on various aspects of women’s health (including risk factors and preventive strategies in order to limit these) were encouraged for this Special Issue.

At the end of July 2020, 60 manuscripts were submitted and, after the peer review process, 23 were accepted for publication in the SI “Potential Risks and Factors of Women’s Health Promotion” of *International Journal of Environmental Research and Public Health*. In particular, 22 original articles (research articles) and one review article were published online upon closure of the Special Issue.

Several topics were examined in this SI, and all manuscripts that were finally published and are available online in open access form are reported in chronological publishing order in Table 1, along with their main characteristics: authorship (first author), topic, location where the research was conducted, period of study, methodology, and the main findings of the study.

A retrospective study conducted in Vietnam from 2014 to 2016, reported an association of one of the most important maternal outcomes, intrauterine growth restriction, in pregnant women that suffer from heart diseases [5].

Wochna and colleagues, in their quasi-experimental field trial, demonstrated the impact of an aqua fitness training program on femur strength index among 18 postmenopausal women [6].

An observational study conducted over a decade (2007–2016) in South Korea investigated the association between gender discrimination in the workplace and pregnancy planning/childbirth experiences among working women and found that gender discrimination, especially in a low/medium income workplace context, is associated with a decreased chance of pregnancy planning/childbirth experience [7].

Alvarez-Villareal M. et al. conducted a qualitative phenomenological study on female patients in a Spanish hospital with chronic kidney diseases (CKD), evidencing that catheters and/or fistulas affected sexual desire and satisfaction [8].

A cohort study conducted in the Netherlands examined the health-related quality of life (HRQoL) of 4312 women after childbirth, identifying multiple determinants of suboptimal physical and mental HRQoL, such as maternal psychopathology after childbirth [9].

An observational study conducted in Jiangxi Province in China that analyzed the utilization of inpatient services for middle-aged and elderly rural females evidenced that hospitalization and late discharge rates were significantly lower for divorced or widowed women than married females [10].

Cernigliaro et al. analyzed the association of individual and contextual inequalities on breastfeeding in the Sicilian region, highlighting that contextual inequalities had a significant association with the risk of not following exclusive breastfeeding in the deprived classes [11].

Another qualitative study conducted in Spain investigated urinary incontinence (UI) as a potential risk factor for women’s health [12]. UI was accompanied by feelings of stress and shame among women; in addition, a lack of information and misconceptions were found [12].

A retrospective observational study in Ecuador demonstrated that the correct use of social networks could help deaf women facing considerable challenges in accessing information on sexual and reproductive health [13].

Antoniou et al. evidenced that domestic violence during pregnancy was more frequent in Greek women with a foreign partner [14]. Unemployed individuals, housewives, and university students faced a higher risk of being abused [14].

In a cross-sectional study on 31,690 Korean adults, Won Kee et al. showed that among women, night eaters had higher odds of depression and depressive symptoms compared with non-night eaters [15].

Middle-aged and elderly females with chronic diseases and non-labor females have a higher probability of getting hospitalized in accordance with a study conducted in a Chinese Province [16].

A cross-sectional study conducted in Vietnam on working hours in relation to work-to-family conflict and burnout found that female employees who work overtime on a voluntary basis are at risk of developing health problems [17].

Vo Van Ha and colleagues demonstrated in a sample of 1617 Vietnamese mothers that resuming physical activity in the early postpartum period is a fundamental weight management strategy [18].

Barcikowska et al. reported that prostaglandins (more than other cytokines) play a major role in the pathomechanism of dysmenorrhea [19].

A quali-quantitative study in a depopulated area of Spain demonstrated that acknowledging the socioeconomic importance of women’s work and adopting measures that facilitate the reconciliation of work and family life were essential measures to consider for women’s well-being and rural development [20].

Rouillon et al. highlighted the importance of health education on endocrine disruptors for pregnant women, taking knowledge, attitudes, and practices into account [21].

An observational study conducted in Hong Kong from 2011 to 2014 evidenced that active play with their preschool children may increase mothers’ physical activity levels [22].

An analysis of intentions to pay for HPV vaccination among women of childbearing age conducted by Thi Thanh Le and colleagues showed that a large majority of women had a firm intention to vaccinate, but after being informed of the current price, the number of respondents who intended to vaccinate dropped to one-fifth [23]. 

An observational study conducted in Dubai found that vasomotor symptoms, fatigue, and obesity were the main symptoms of psychological distress among menopausal women [24].

A multicenter Italian cross-sectional survey on knowledge and attitudes regarding HPV infection and prevention among healthcare workers showed overall positive attitudes towards HPV burden and prevention tools, although knowledge was largely suboptimal [25].

Environmental tobacco smoke exposure during pregnancy, and not air pollution, decreased birth length and is an independent risk factor for fetal growth restriction according to data reported in a longitudinal study conducted in Poland [26].

Finally, a cross-sectional study conducted on first-grade secondary schools in Sicily that evaluated the knowledge, attitudes, and willingness to vaccinate against HPV among preadolescents demonstrated a good level of knowledge and attitudes, and that willingness to receive HPV vaccination significantly increased after vaccination counselling interventions at school [27].

In conclusion, one of the main challenges facing future public health is the improvement of preventive measures in order to reduce the burden of acute and chronic diseases among the general female population [2].

Women’s health promotion could be supported throughout strong multidisciplinary alliances between healthcare professionals, providing evidence-based data on preventive strategies adopted to counteract cervical cancer (such as HPV vaccination and cervical cancer screening), the breastfeeding attitudes of healthcare workers and mothers, pregnancy outcomes (with a specific focus on women with different deprivation levels), and the impact and effectiveness of communication strategies, especially for high-risk groups of women [1].

## Figures and Tables

**Table 1:** Description of the manuscripts published in the Special Issue “Potential Risks and Factors of Women’s Health Promotion” in chronological publishing order.

Authorship	Research Topic	Location	Period of Study	Study Methodology	Main Findings
Nguyen Manh T. et al. [5]	Maternal outcomes in pregnant women suffering from heart disease	Hanoi, Vietnam	2014–2016	Retrospective study	Heart diseases were significantly associated with intrauterine growth restriction.
Wochna K. et al. [6]	Impact of aqua fitness training on bone tissue	Poznan, Poland	2018–2019	Quasi-experimental interventional trial	Aqua fitness training program caused favorable changes in femur strength index in postmenopausal women.
Kim J.H. et al. [7]	Effects on pregnancy outcomes of gender discrimination in the workplace	South Korea	2007–2016	Longitudinal study	Gender discrimination in the workplace is associated with decreased odds of pregnancy planning/childbirth experience among working South Korean women, especially for low/medium income.
Alvarez-Villareal M. et al. [8]	Body changes and sexual drive in female patients with chronic kidney diseases (CKD)	Madrid, Spain	2017–2018	Qualitative phenomenological study	Catheter and/or the fistula triggered sexuality changes among CKD women, affecting sexual desire and satisfaction.
Bai G. et al. [9]	Determinants of health-related quality of life (HRQoL) after childbirth	Netherlands	2002–2018	Cohort study	Identification of multiple determinants of suboptimal physical and mental HRQoL after childbirth.
Wen X. et al. [10]	Hospitalization service utilization between permanent and migrant females in rural regions	Jiangxi Province, China	2006–2014	Prospective observational study	For both female permanent residents and migrants, the older their age, the higher their hospitalization rate as related mainly to chronic diseases.
Cernigliaro A. et al. [11]	Impact of individual/context inequalities on breastfeeding	Sicilian Region, Italy	2017–2018	Prospective cohort study	Context inequalities had a significant association with the risk of not following exclusive breastfeeding in the deprived classes.
Pintos-Diaz M.Z. et al. [12]	Urinary incontinence (UI) and potential risks for women’s health	Madrid, Spain	2015–2017	Qualitative descriptive study	UI was accompanied by stress and shame among women, and lack of information and misconceptions were found.
Robles-Bykbaev Y. et al. [13]	Access of information on sexual and reproductive health among deaf women in Ecuador	Ecuador	2017–2018	Retrospective observational study	Correct use of social networks could help deaf women facing considerable challenges in education and healthcare.
Antoniou E. et al. [14]	Domestic violence during pregnancy	Athens (Greece)	2019	Longitudinal study	Pregnant women with a foreign partner, unemployed individuals, housewives, and university students faced a higher risk of being abused.
Won Lee K. et al. [15]	Association of night eating with depression and depressive symptoms	South Korea	2008–2013	Cross-sectional study	In women, night eaters had higher rates of depression and depressive symptoms compared with non-night eaters.
Wen X. al. [16]	Utilization of inpatient services for middle-aged and elderly rural females	Jiangxi Province, China	2006–2014	Prospective observational study	Hospitalization and late discharge for divorced or widowed females was significantly lower than for married women.
Huang S.L. et al. [17]	Working hours in relation to work-to-family conflict and burnout	Taiwan	2013–2014	Cross-sectional study	Female employees who work overtime on a voluntary basis are at risk of health problems.
Vo Van Ha A. et al. [18]	Postpartum physical activity and weight retention	Vietnam	2015–2017	Prospective cohort study	Resuming physical activity in the early postpartum period is a fundamental weight management strategy.
Barcikowska Z. et al. [19]	Inflammatory markers in dysmenorrhea	Poland	2020	Review	Prostaglandins (more than other cytokines) play a major role in the pathomechanism of dysmenorrhea.
Cobano-Delgado V. et al. [20]	Women’s well-being and rural development in depopulated Spain	Spain	2017–2018	Qualitative and quantitative study	Acknowledging the socioeconomic importance of women’s work and adopting measures that facilitate the reconciliation of work and family life is essential.
Rouillon S. et al. [21]	Educate pregnant women about endocrine disruptors	Poitiers, France	2014–2020	Cross-sectional study and randomized controlled intervention trial	Health education on endocrine disruptors should be conducted for pregnant women, taking knowledge, attitudes, and practices into account.
Carver A. et al. [22]	Socioeconomic status and physical activity among mothers of young children	Hong Kong	2011–2014	Observational study	Encouraging active play with their preschoolers may increase mothers’ physical activity levels.
Thi Thanh Le X. et al. [23]	Intention to pay for HPV vaccination among women of childbearing age	Hanoi, Vietnam	2016	Cross-sectional study	Respondents expressed a firm intention to vaccinate. However, after being informed of the current price the number of respondents who intended to vaccinate dropped to one-fifth.
Mohammed Ali A. et al. [24]	Psychological climacteric symptoms and attitudes toward menopause	Dubai, United Arab Emirates	2018	Observational study	Psychological distress among menopausal women manifests itself with vasomotor symptoms, fatigue, and obesity.
Trucchi C. et al. [25]	Healthcare professionals’ knowledge and attitudes regarding HPV infection and prevention	Liguria and Apulia Regions (Italy)	2017–2018	Cross-sectional study	Although healthcare workers showed overall positive attitudes towards HPV burden and prevention tools, knowledge was largely suboptimal.
Chen M.M. et al. [26]	Environmental tobacco smoke and air pollution on fetal growth	Poland	2017–2019	Prospective longitudinal study	Environmental tobacco smoke exposure decreased birth length and is an independent risk factor for fetal growth restriction.
Costantino C. et al. [27]	Knowledge, attitudes, and willingness to vaccinate against HPV among preadolescents	Sicilian Region (Italy)	2017–2019	Cross-sectional study	Willingness to receive HPV vaccination significantly increased after vaccination counselling interventions at school.
